# Spatial mapping of juxtacrine axo-glial interactions identifies novel molecules in peripheral myelination

**DOI:** 10.1038/ncomms9303

**Published:** 2015-09-18

**Authors:** Y. Poitelon, S. Bogni, V. Matafora, G. Della-Flora Nunes, E. Hurley, M. Ghidinelli, B. S. Katzenellenbogen, C. Taveggia, N. Silvestri, A. Bachi, A. Sannino, L. Wrabetz, M. L. Feltri

**Affiliations:** 1 Hunter James Kelly Research Institute, Department Biochemistry, University at Buffalo, Buffalo, New York 14203, USA; 2 Division of Genetics and Cell Biology, San Raffaele Hospital, Milano 20132, Italy; 3 Department of Molecular and Integrative Physiology, University of Illinois and College of Medicine, Urbana Illinois 61801, USA; 4 Division of Neuroscience, San Raffaele Hospital, Milano 20132, Italy; 5 Department of Neurology, School of Medicine and Biomedical Sciences, University at Buffalo, Buffalo, New York 14203, USA; 6 Department of Engineering for Innovation, University of Salento, Lecce 73100, Italy

## Abstract

Cell–cell interactions promote juxtacrine signals in specific subcellular domains, which are difficult to capture in the complexity of the nervous system. For example, contact between axons and Schwann cells triggers signals required for radial sorting and myelination. Failure in this interaction causes dysmyelination and axonal degeneration. Despite its importance, few molecules at the axo-glial surface are known. To identify novel molecules in axo-glial interactions, we modified the ‘pseudopodia’ sub-fractionation system and isolated the projections that glia extend when they receive juxtacrine signals from axons. By proteomics we identified the signalling networks present at the glial-leading edge, and novel proteins, including members of the Prohibitin family. Glial-specific deletion of Prohibitin-2 in mice impairs axo-glial interactions and myelination. We thus validate a novel method to model morphogenesis and juxtacrine signalling, provide insights into the molecular organization of the axo-glial contact, and identify a novel class of molecules in myelination.

## Abstract (web summary)

Neuron–glia interactions are critical in the nervous system, where they result in the extension of glial pseudopodia. Poitelon *et al*. isolate these protrusions using an *in vitro* assay, and, by characterising their proteomes, identify Prohibitin-2 as a regulator of myelination.

## Graphical abstract

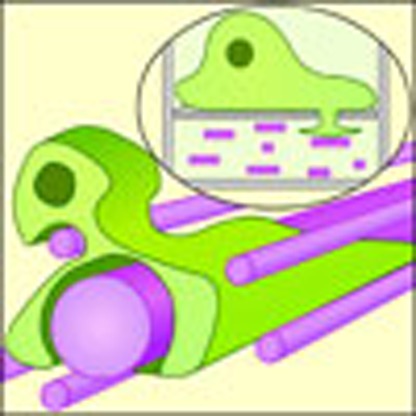

Myelin is required for fast conduction of neural impulses, preserves axons, and is implicated in demyelinating and neurodegenerative diseases^1^. The core of this function lies at the polarized surface of contact between myelin-forming glia and axons. Because this surface lies beneath a series of concentric inward wraps of myelin, it is inaccessible to biochemical isolation, making the studies of this crucial nervous system apposition arduous. Indeed, only few molecules have been identified in this location. More generally, compartmentalization of signalling events is crucial for glia, neurons and other polarized cells, and cell–cell interactions are at the basis of morphogenesis and are required for the function of all tissues. Only a few tools are available to study these events in specific subcellular domains.

Insights into the spatial organization of signalling networks have been obtained using the pseudopod subcellular fractionation system, in cells responding to chemotactic (soluble) or haptotactic (extracellular matrix) stimuli^2^,^3^. In this system^2^, cells are placed in a chamber with a microporous membrane, and extend pseudopodia in response to stimuli in the bottom chamber. Pseudopods can be imaged and isolated from the cell body, which remains on the top chamber due to the size of the nucleus. However, this method has not yet been applied to juxtacrine signalling.

Here we sought to adapt the pseudopod system to the study of cell–cell interactions in the nervous system. Our goal is to identify signalling events, molecular networks and novel molecules specifically at the axo-glia interface. Schwann cells (SC) make myelin in peripheral nerves, in response to contact with axons and the basal lamina (reviewed in ref. 4), making them excellent instruments to test if pseudopod sub-fractionation can model juxtacrine signalling. To ask whether this system was suitable to study signals between axons and glia, we replaced soluble stimuli with neuronal cell membranes, to mimic cell–cell contact, and isolated the cytoplasmic processes (pseudopods) that Schwann cells extend to contact axon membranes. By proteomic analysis we identified proteins that partitioned in pseudopods uniquely after axonal signals, including novel proteins and proteins known to localize at the Schwan cell–axon interface *in vivo*. We also identified the major signalling networks induced by Schwann cell–axonal contact, and a novel protein family, Prohibitins, which we show is required for appropriate axo-glial interactions *in vivo*. This validation confirms that we were able to successfully model complex morphogenetic cell–cell interactions. This novel method opens the possibility to map subcellular events that occur during the interactions between neurons and other glial cells, or between any cells.

## Results

### SC extend pseudopods in response to chemotactic stimuli

**Figure 1:**
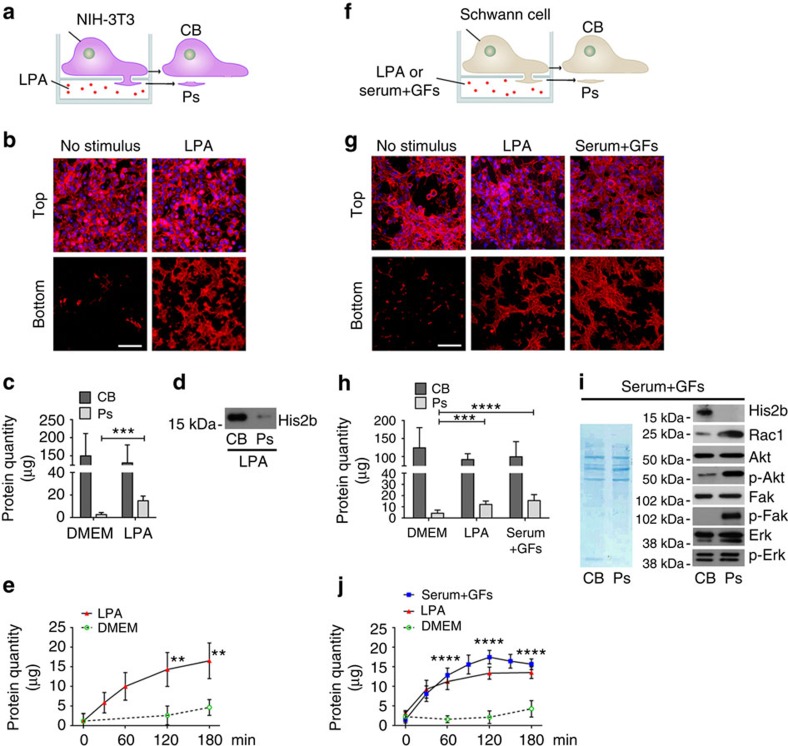
Schwann cells extend pseudopods in response to a soluble chemoattractants. (**a**,**f**) Schematic representation of NIH 3T3 (to the left) and Schwann cell (to the right) extending pseudopod towards a chemoattractant gradient of either lysophosphatidic acid (LPA) or fetal calf serum and growth factors. The cell body (CB) can be physically separated from the pseudopods (Ps). (**b**,**g**) Confocal images of NIH 3T3 (**b**) and Schwann cell (**g**) bodies (top) or pseudopods (bottom) in response to the indicated stimuli. Cells were stained for F-actin with TRITC-phalloidin and with DAPI to visualize nuclei. A small amount of random, non-polarized pseudopods are present in the absence of stimulus. (**c**,**h**) Quantification by BCA of the protein content of NIH 3T3 (**c**) and Schwann cell (**h**) pseudopods and cell bodies, after induction with the indicated stimuli. (**d**,**i**) Western blots from NIH 3T3 (**d**) and Schwann cell (**i**) protein lysates from pseudopods and cell bodies, after induction by the indicated stimuli. Equal amount of proteins were separated by SDS/PAGE, probed for the indicated proteins and stained with amido black. (**e**,**j**) Growth kinetics of NIH 3T3 (**e**) and Schwann cell (**j**) pseudopods. The amount of proteins was determined for the indicated times after addition of the stimuli. Error bars indicate s.d. *n*=3 independent experiments in **e**,**j**, *n*=5 independent experiments in **c**,**h**. Statistical analyses were performed using *t*-test (**c**), one-way ANOVA (**h**) and two-way ANOVA (**e**,**j**). **P<0.01, ***P<0.001 and ****P< 0.0001. Scale bar, 40 μm.

Several cell types such as fibroblasts (NIH3T3) *in vitro* respond to gradients of certain chemoattractants (for example, lysophosphatic-lysophosphatidic acid (LPA), but not insulin) by directional cell migration^2^. The initial step is the extension of a polarized protrusion (‘pseudopod’) that can be isolated by placing the cells on top of a Boyden chamber with microporous filters (3-μm pores) and the chemoattractant on the bottom^2^ (Fig. 1a). Cell bodies and pseudopods can then be separated.

To determine if primary Schwann cells respond like other cells, we compared their response (Fig. 1f–j) to that of NIH3T3 cells (Fig. 1a–e) to LPA, or to a combination of fetal calf serum, forskolin and growth factors. By staining the top or the bottom of the membrane with phalloidin and DAPI, and imaging with confocal microscopy focused sequentially above and below the membrane, we showed that both primary Schwann cells and NIH3T3 cells extended pseudopods in response to the chemoattractants, but not to DMEM alone (Fig. 1b,g). As expected, nuclei were detected solely on the upper surface, confirming that only pseudopods protruded through the small pores. Proteins from the cell bodies or pseudopods can be solubilized independently and quantified or analysed. Polarization was confirmed biochemically by showing that nuclear histones were present mostly in the cell body fraction (Fig. 1d,i). Protein quantification after 2 h of exposure to LPA or serum and growth factors confirmed that significantly more proteins were extracted from the pseudopod fraction in the presence of the chemoattractants (Fig. 1c,h). We next asked if the kinetics of pseudopod growth were similar in the two cell types. The extension of pseudopods in both Schwan cells and NIH3T3 cells was detected as early as 30 min after exposure to chemoattractants, and pseudopod growth reached a plateau after 2 h (Fig. 1e,j), similar to previous reports. Removal of the chemoattractant reversed the polarization of the Schwann cells and induced pseudopod retraction (Supplementary Fig. 1), confirming that the formation of pseudopods is an active and reversible process^2^.

Previous studies showed that the molecular machinery required for the extension of pseudopods resembles that required for extension of a leading edge in polarizing cells at the beginning of migration^2^, including regulators of the actin cytoskeleton. We thus wished to confirm that Schwann cell pseudopods contained regulators of actin polymerization, and molecules required for formation of Schwann cell protrusions. For example, Rac1, and Fak are required *in vivo* for Schwann cells to form polarized lamellipodia around axons and to begin myelination^5^,^6^ and p-Akt is associated with the onset of myelination^7^; in contrast p-Erk is not required for initial wrapping, but for addition of subsequent myelin layers^8^,^9^. Indeed we found that Rac1, p-Fak and p-Akt, but not p-Erk, were enriched in pseudopods (Fig. 1i).

Cells can polarize and migrate also towards haptotactic stimuli, like polymerized extracellular matrix, and this can also be mimicked in the pseudopod system^3^. Accordingly, Schwann cells extended pseudopods in response to Laminin polymerized on the lower membrane and the Laminin receptors β-Dystroglycan redistributed to these pseudopods (Supplementary Fig. 2).

These results demonstrate that Schwann cells respond to soluble or haptotactic physiological stimuli by extending polarized pseudopods, which contain the appropriate molecular machinery.

### SC extend pseudopods in response to neuronal membranes

It is established that contact between Schwann cells and axons is strictly required for induction of myelination. Neuronal membrane preparations have been used as a model for axonal contact itself^10^,^11^. Thus, we asked if Schwann cells could extend polarized pseudopods in response to juxtacrine signals from neuronal membranes, and if the Schwann cell response to neuronal membranes was specific and unique.

**Figure 2:**
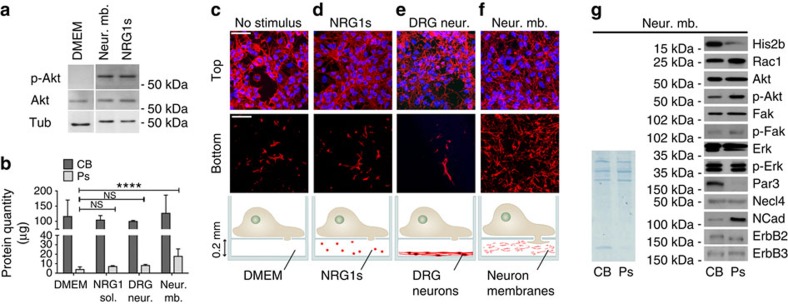
Schwann cells extend pseudopods selectively in response to juxtacrine-like neuronal signals. (**a**) Activation of Akt in Schwann cells by neuronal membrane suspensions. Neuronal membranes were added to Schwann cells, protein lysates were probed for p-Akt, Akt and β-Tubulin. Soluble Neuregulin1 was used as a positive control. (**c**–**f**) Confocal images of Schwann cell pseudopods and cell bodies in response to the indicated stimuli in the bottom chamber. Cells were stained for F-actin with TRITC-phalloidin and DAPI. (**c**) No stimulus (DMEM). (**d**) Soluble neuregulin 1 (NRG1s). (**e**) DRG neurons in defined media plated on the bottom chamber, at 0.2-mm distance below the microporous surface. (**f**) DRG neurons were solubilized and the membrane suspension was added to the bottom chamber. Only this latter condition caused the extension of polarized pseudopods. (**b**) Quantification by BCA of the protein content of Schwann cell pseudopod and cell bodies in the conditions indicated. Error bars indicate s.d. *n*=5 independent experiments. Statistical analyses were performed using one-way ANOVA, ****P< 0.0001. (**g**) Western blots from Schwann cell pseudopods and cell body protein lysates, after induction by neuronal membranes. Equal amount of proteins were separated by SDS/PAGE probed for the indicated proteins and stained with amido black. Scale bar, 40 μm.

We prepared neuronal membranes from isolated dorsal root ganglia (DRG) neurons, and confirmed that they were biologically active by showing that they induced Akt phosphorylation in Schwann cells (Fig. 2a). The key juxtacrine signal on axons is Neuregulin 1-type III, which is required for Schwann cell myelination^12^,^13^, while soluble Neuregulin 1-type 1 cannot promote myelination unless it is presented at high concentration or after injury^14^,^15^. Consistent with these published data, when we placed soluble Neuregulin 1-type I on the bottom chamber, Schwann cells failed to extend pseudopods (Fig. 2b,d). Similarly, when we plated DRG neurons on the bottom chamber, at a distance (0.2 mm) from the Schwann cells that precludes contact, the physical separation prevented formation of pseudopods (Fig. 2b,e). In contrast, when we filled the bottom chamber with a suspension of neuronal membranes, such that integral proteins in neuronal membranes could contact the Schwann cells, Schwann cells responded by making polarized protrusions (Fig. 2b,f). These data suggest that the formation of Schwann cell protrusions requires neuronal contact, like myelination itself, and they are consistent with the notion that soluble signals from neurons are not sufficient to induce myelination^12^. Three-dimensional (3D) reconstruction and volume rendering showed that most cells responded by extending pseudopods, and that pseudopods were polarized (that is, extended only through the pores towards the neuronal membranes; Supplementary Fig. 3, Supplementary Movie 1). Importantly, membrane suspensions prepared from non-neuronal cells did not induce the extension of Schwann cell protrusions (Supplementary Fig. 4). Thus, only the direct contact between Schwann cells and neuronal membranes promotes the extension of Schwann cell pseudopods, supporting that they may represent a model of the first polarization events leading to myelination.

As before, we confirmed that molecules required for extension of membrane protrusions, migration and myelination such as Rac1, p-Fak and p-Akt partitioned in the pseudopods induced by neuronal membranes, while nuclear histones partitioned preferentially in the cell bodies (Fig. 2g). We next asked whether Schwann cell pseudopods contained molecules located at the glial surface that faces axons. Remarkably, only few molecules at this important site are known. Among them, *N*-cadherin is polarized immediately to sites of axo-glial contact, where it recruits Par3; Necl4 localizes at the axo-glial surface in mature fibers, and the receptor ErbB2/3 should localize near axons to interact with axonal Neuregulins^16^,^17^,^18^,^19^. Notably, only *N*-cadherin, that mediates the first axonal contact, was clearly enriched in pseudopods, whereas ErbB receptors and Necls were present in both fractions, and Par3 was only detectable in cell bodies. This suggests that full polarization requires either >2 h after axonal contact or a second polarizing event, likely from the basal lamina. Alternatively, it is possible that post-translational modifications that activate these molecules occur preferentially in pseudopods. For example, it will be interesting in the future to determine whether ErbB2 and ErbB3 phosphorylation occurs mainly in pseudopods. Finally, pseudopods did not contain the axonal specific molecules Caspr and Contactin-1 (Supplementary Fig. 5), indicating that neuronal membranes did not contaminate the pseudopod proteome. These results demonstrate that Schwann cells respond to juxtacrine signals in neuronal membranes by producing polarized pseudopodia that mimic the early cellular protrusions of intact neuro-glial systems. Thus the pseudopod system can model specific cell–cell interactions.

### Pseudopods induced by neurons contain a specific proteome

**Figure 3:**
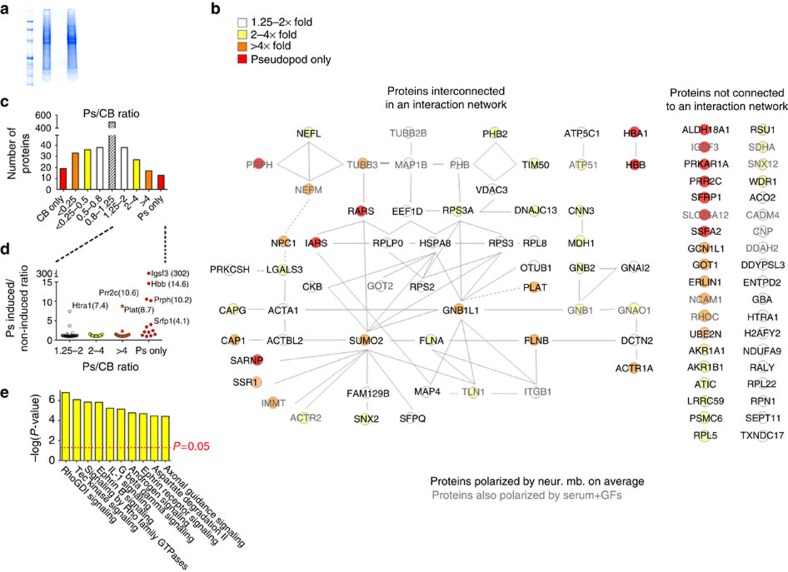
Proteins enriched in pseudopods after induction by neuronal membranes. (**a**) Coomassie blue staining shows that equal amounts of lysates from Schwann cell bodies or pseudopods were separated on SDS/PAGE and used for proteomic studies. (**b**) Interaction network generated by the Ingenuity IPA software of proteins enriched in Schwann cell’s pseudopod (Ps/CB ratio>1.25) after induction by neuronal membranes (Ps induced by neurons/Ps non induced >1). The signalling network consists of 95 proteins that are described in Supplementary Data 2. Proteins are named by their official gene symbol according to Entrez Gene (www.ncbi.nlm.nih.gov). The relative protein enrichment in pseudopods versus cell bodies (Ps/CB ratio) is colour coded according to the colour scale at the top of the figure. Proteins that were also enriched in pseudopods by serum and growth factors (Supplementary Data 3) have been given in grey. The references supporting the protein–protein interactions are listed in Supplementary Data 6. (**c**) Distribution of the number of Schwann cell proteins that were enriched in pseudopods based on the Ps/CB ratio. (**d**) Distribution of the number of proteins enriched by neuronal membranes specifically, based on the Ps induced by neurons/Ps non-induced ratio (second enrichment criteria). (**e**) Protein ontology analysis of the pathways most enriched in Schwann cells pseudopods (10 out of 162 shown, for complete listing, see Supplementary Data 5).

Having shown that Schwann cells respond specifically to juxtracrine neuronal signalling by producing appropriate polarized protrusions, we next determined the proteome of the pseudopods and compared it to that of cell bodies. We analysed equal amounts of pseudopod and cell body lysates (Fig. 3a, Supplementary Fig. 5a) and quantified the relative abundance of a given protein in the lysates in the induced (neuronal membranes) versus non-induced (DMEM) condition, by stable isotope labelling (SILAC), coupled with liquid chromatography–tandem mass spectrometry (LC–MS/MS). We reliably identified 714 proteins, 605 of which were present in neuronally induced pseudopods (Supplementary Data 1). Of these, 176 proteins were enriched in pseudopods (as compared to cell bodies: Ps/CB ratio>1.25, Fig. 3c). Among these, 95 proteins were also relatively increased in pseudopods after induction with neuronal membranes (as compared to DMEM: Ps induced by neurons/Ps non induced >1, Fig. 3b,d and Supplementary Data 2).

Formation of polarized protrusions in any cells requires the cytoskeleton. Accordingly, the largest group of proteins enriched in pseudopods and increased during neuronal contact was associated with the cytoskeleton (19%; Supplementary Data 2). However, we wished to ask whether neuronal membranes also induced a specific response in Schwann cells, given the unique molecular and geometrical relationships between the two cells. To distinguish between molecules induced by any chemotactic, promigratory stimulus and molecules induced specifically by neuronal membranes, we compared the proteome of Schwann cell pseudopods generated by neuronal membranes (Supplementary Data 1) with that generated in response to fetal calf serum plus growth factors (Supplementary Data 3). Notably, we identified only 23 proteins that were enriched by both stimuli, representing 24% of the proteins enriched in pseudopods by neuronal membranes (Fig. 3b, indicated in grey). The remaining 76% of the proteins were enriched exclusively by neuronal membranes (Fig. 3b, indicated in black, Supplementary Data 4). This suggests that signalling by neuronal membranes elicits the redistribution not only of proteins required for the formation of cellular protrusions, it also induces a larger and unique set of specific proteins.

We next asked whether the proteome that varied in response to neuronal membranes included novel proteins, and proteins already known to function in myelination, both were present. Among the 95 proteins that increased in response to interaction with neuronal membranes, we found Integrin β1, Necl4 and cAMP-dependent protein kinase type I—α regulatory subunit—that are required for Schwann cell–axon interactions *in vivo*^20^,^21^,^22^, whereas N-CAM mediates homotypic axon-Schwann cell interactions *in vitro*^23^. Other proteins are implicated in oligodendrocyte myelination and integrity *in vivo*, such as Galectin 3^24^ and Niemann–Pick C1 (NP–C)^25^.

Overall, these results reveal that contact with neuronal membranes induces significant Schwann cell polarization. Remarkably, 76% of the proteins identified were uniquely redistributed after induction with neuronal membranes, but not after exposure to serum and growth factors, indicating that juxtacrine signals from neurons induce a specific set of proteins to redistribute in Schwann cells. Finally, the presence of molecules known to be important in axo-glial interactions indicates that our method is successful in the identification of proteins relevant to axo-glial contact and myelination.

### Novel proteins and signalling networks in pseudopods

We next characterized the interaction network of polarized protrusions in Schwann cells and searched for novel proteins. We used Ingenuity Pathways Analysis to find canonical pathways that were involved, and to represent the protein-protein interactome (based on the Ingenuity Knowledge Base). Strikingly, many of the proteins that were polarized by axonal contact were interconnected in networks (Fig. 3b) suggesting that they are functionally related. The pathways most enriched in the pseudopods included both novel signalling networks and proteins already shown to be important. For example, Rho GTPases and Rho GDI, Tec Kinase and G Beta Gamma signalling (Fig. 3e, Supplementary Data 5) were the pathways most represented. β1 integrin and RhoC were constituents of these three pathways (Supplementary Data 5), suggesting a potentially important role for RhoC, given that β1 integrin and other small Rho GTPases and effectors (Rac1, Cdc42, Rock and Mlck) are required for axo-glial interactions^6^,^21^,^26^,^27^,^28^. Similarly, 9 out of the 10 most abundant pathways shared the heterotrimeric G proteins encoded by *Gnb1*, *Gnb2*, *Gnb2L1*, *Gnao1* and *Gnai2* (Supplementary Data 5), which expand the notion that G-protein signalling is a major contributor of radial sorting and myelination^29^.

We also noted that three out of the seven pseudopod proteins that were most induced by neuronal membranes as compared to DMEM (above 5 × fold, Fig. 3d) were secreted (that is, Secreted frizzled-related protein 1, a Wnt inhibitor; Htra1 and Tissue plasminogen activator (tPA) encoded by *Plat*). Interestingly, among these secreted molecules Wnt signalling is important during Schwann cell development^30^, and tPA is important during axonal regeneration^31^.

We also identified three proteins with a Prohibitin homology domain: Prohibitin-1, Prohibitin-2 and Erlin-1. This domain is found in a small subset of proteins in higher eukaryotes: Prohibitins, Erlins, Stomatins, Flotillins and Podocin (http://smart.embl.de/). Originally described and characterized as mitochondrial inner-membrane proteins^32^, Prohibitins have pleiotropic effects. They localize at the plasma membrane, where they interact with various receptors and activate the Ras–Raf signalling pathway^33^; in the cytoplasm, where they interact with mTOR^34^; or in the nucleus, where they are Akt substrates^35^ and repress transcription of Yy1^36^. As all of these latter molecules are involved in myelination, we asked if Prohibitins engaged in axo-glial interactions.

### Subcellular localization of prohibitins

**Figure 4:**
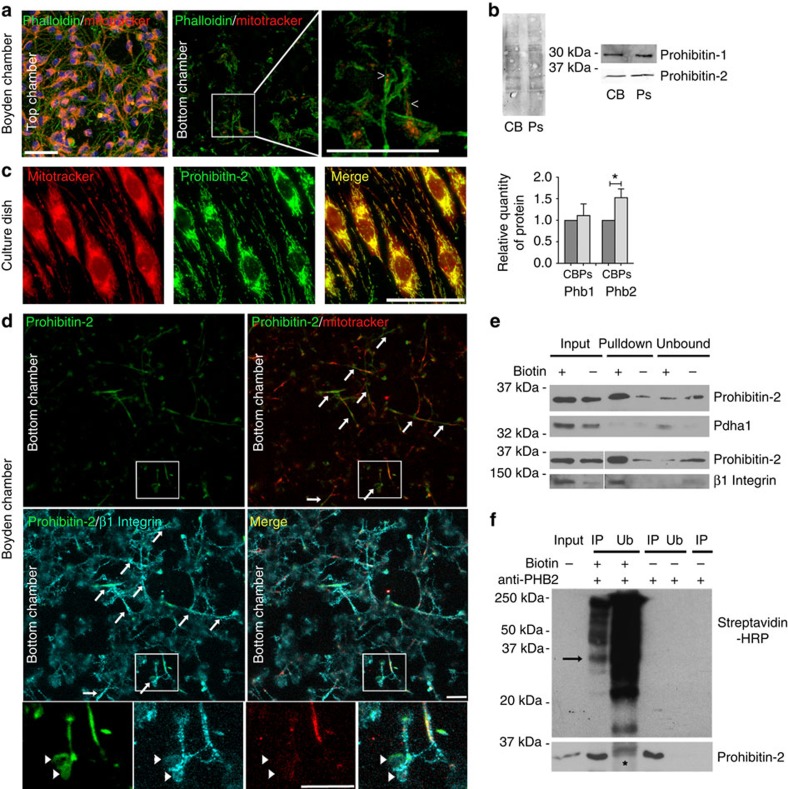
Localization of Prohibitins in Schwann cells. (**a**) Schwann cell pseudopods contain some mitochondria. Mitotracker staining (red) in Schwann cells on the top (cell bodies) or bottom (pseudopods) of the chamber, after induction with neuronal membranes. Cells were co-stained with FITC-phalloidin and DAPI. The white box is enlarged in the right panel. Arrowheads indicate mitochondria in pseudopods. (**b**) Western blots of protein lysates from Schwann cell pseudopods and cell bodies, after induction by neuronal membranes. Equal amount of proteins were separated by SDS/PAGE, probed for Prohibitin-1 and Prohibitin-2 and stained with amido black. Prohibitin-2 was enriched in the pseudopod fraction. Error bars indicate s.d. *n*=3 independent experiments. Statistical analyses were performed using *t*-test. *P< 0.05. (**c**) Immunolocalization of Prohibitin-2 in cultured Schwann cells. Cells were stained for Prohibitin-2 (green) and Mitotracker (red). Prohibitin-2 is detected mostly in mitochondria. (**d**) Immunolocalization of Prohibitin-2 in Schwann cell’s pseudopods, on the bottom of the Boyden chamber, after induction with neuronal membranes. Cells were stained for Prohibitin-2 (green), Mitotracker (red) and β1-Integrin (cyan). Prohibitin-2 is found in pseudopods, where it partially co-localizes with mitotracker and in some areas co-localizes with β1-Integrin. Arrows show areas where Prohibitin-2 co-localizes with β1-Integrin, but not mitotracker. The white box is enlarged in the bottom panels, and shows a Schwann cell lamellipodia-like protrusion stained for β1 integrin and Prohibitin-2, but not mitotracker (arrowheads). (**e**) Streptavidin-pull-down of biotinylated cell-surface proteins in Schwann cells. Equal amount of proteins were pulled-down by Streptavidin–Sepharose, separated by SDS/PAGE and probed for Prohibitin-2 and Pdha1 (mitochondrial marker) or Prohibitin-2 and β1-Integrin (plasma membrane marker). Prohibitin-2 and β1-Integrin, but not Pdha1, are present in the biotin fraction. Probing for β1-Integrin and Pdha1 were performed on two different blots. (**f**) Immunoprecipitation of Prohibitin-2 in Schwann cells after biotinylation of cell-surface proteins. Equal amount of proteins were precipitated by Prohibitin-2 antibody, separated by SDS/PAGE and stained for Streptavidin-HRP then Prohibitin-2. A biotionylated protein at the size of Prohibitin-2 is present (35 kDa, arrow). Other biotinylated proteins co-immunoprecipitate with Prohibitin-2. The band in the unbound fraction (asterisk) corresponds to the Streptavidin–HRP detection performed first. (IP: immunoprecipitation, Ub: unbound fraction). Scale bars, 40 μm (**a**,**c**); 10 μm (**d**).

Among the 95 proteins that were polarized and enriched in protrusions by neuronal membranes, 4 were predicted to be secreted, 16 transmembrane or membrane associated, 53 cytoplasmic, 19 in organelles (mitochondrial, ER, peroxisome and lysosomes) and 3 nuclear (Supplementary Data 2). Because 12% of these proteins were identified as mitochondrial, including Prohibitins, we asked whether mitochondria were present and enriched in pseudopods. We stimulated Schwann cells with neuronal membranes and stained them with phalloidin and mitotracker. We detected some mitochondria in the pseudopods, although the majority of mitochondria were found in cell bodies (Fig. 4a).

To characterize the localization of Prohibitins in Schwann cells, we first confirmed that Prohibitin-1 and -2, were present in pseudopods by infrared-based western blot analysis. Both proteins were present in pseudopods, and we could confirm that Prohibitin-2, was more abundant in pseudopods (2.8 Ps/CB ratio by SILAC mass spectrometry; Fig. 4b). While we could not detect a significant enrichment of Prohibitin-1, this is probably due to the poor quantitative sensitivity of western blot in the range of PhB1 enrichment (1.3 Ps/CB ratio by SILAC mass spectrometry).

We next asked whether we could detect Prohibitins in Schwann cell mitochondria and pseudopods, and we focused on the more polarized Prohibitin-2. By immunohistochemistry, the majority of Prohibitin-2 in Schwann cells cultured on poly-L-lysine co-localized with mitochondria (Fig. 4c). When we exposed Schwann cells to neuronal membranes in the Boyden chamber, Prohibitin-2 was readily detected in pseudopods, where it still colocalized with mitochondria, but also with the membrane protein β1 integrin in small lamellipodia (Fig. 4d).

Because Prohibitins are exposed to the external membrane of activated T cells^37^ we asked whether Prohibitins were also present at the external surface of Schwann cells. We biotinylated Schwann cells, and followed by either streptavidin pull-down and blotting with anti-Prohibitin-2 antibodies (Fig. 4e) or by Prohibitin-2 immunoprecipitation and blotting with Streptavidin-HRP (Fig. 4f). In both cases, Prohibitin-2 was found together with biotinylated proteins of Schwann cell plasma membrane. β1 integrin, a membrane protein, behaved like Prohibitin-2 (Fig. 4e), whereas the exclusively mitochondrial protein Pdha-1 could not be pulled-down by biotin–streptavidin (Fig. 4e). These data indicate that a fraction of Prohibitin-2 is either present on the Schwann cell surface, or interacts with proteins present on Schwann cell surface. However, this fraction must be small, because by immunocytochemistry the majority of Prohibitin-2 was detected in mitochondria in cultured Schwann cells (Fig. 4a). Thus, Prohibitins localize to pseudopods in Schwann cells, where they are present both in mitochondria and at the cell membrane.

### Prohibitins are necessary for myelination *in vitro*

**Figure 5:**
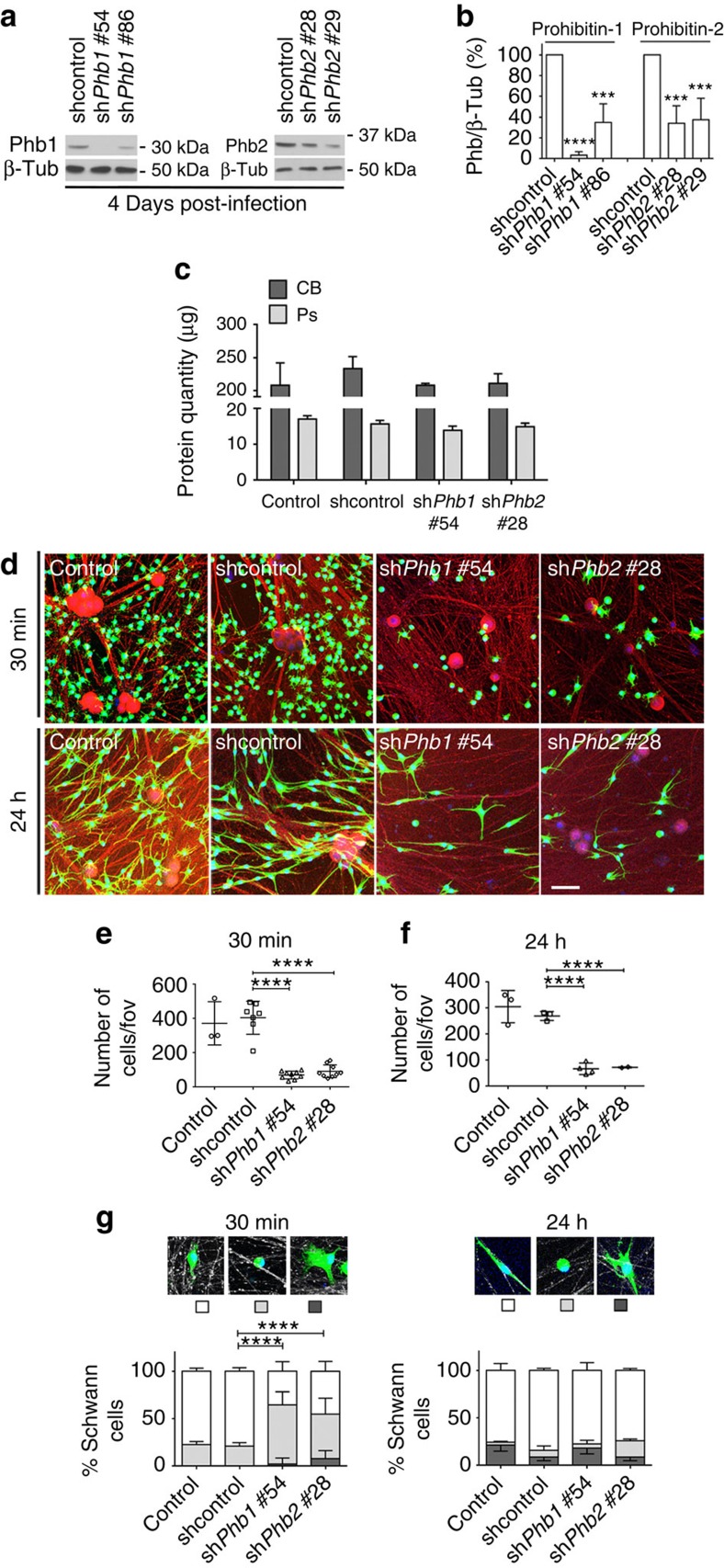
Prohibitins participate in the initial axoglial contact. (**a**) Silencing of *Phb1* or *Phb2* in Schwann cells. Protein lysates of Schwann cells after transfection with sh*RNA* for either *Phb1* or *Phb2* were blotted and probed for Prohibitin-1, Prohibitin-2 and β-Tubulin. (**b**) Quantification of Prohibitin-1 and Prohibitin-2 protein levels in Schwann cells silenced for either *Phb1* or *Phb2*. (**c**) The amount of proteins in Schwann cell pseudopod does not change after Prohibitin silencing. (**d**) Early interactions between Schwann cells (stained with cell tracker, green) and axons (immunostained for NFH, red) are modified by Prohibitin silencing. Schwann cells (100,000) were seeded on neurons for 30 min or 24 h. Ascorbic acid was added after 8 h in the 24-h condition. (**e**,**f**) Number of Schwann cells in contact with neurons after 30 min (**e**) or 24 h (**f**). (**g**) Classification of Schwann cell morphology based on their relationship with axons. Axons are pseudocoloured in white. At 30 min, white indicates a Schwann cell contacting axons, light grey indicates a round Schwann without any protrusion, and dark grey indicates a Schwann cell with a large cytoplasm not extending towards an axon. We observed a significant reduction of Schwann cells contacting axons (white) when Prohibitin-1 or Prohibitin-2 are silenced. Large Schwann cell not extending towards axons were only observed after silencing of Prohibitins. At 24 h, white indicates a bipolar Schwann cell extending along an axon, light grey indicates a round Schwann without any protrusion and dark grey indicates a Schwann extending in more than two directions towards several axons. Error bars indicate s.d. *n*=3 independent experiments in **b**,**c**; *n*= 3 coverslips from three independent experiments in **e**–**g**. Statistical analyses were performed using one-way ANOVA. ***P<0.001, ****P<0.0001. Scale bar, 75 μm.

To determine the function of Prohibitins in Schwann cells, we first asked if silencing Prohibitins would affect the extension of Schwann cell pseudopods and their interaction with neurons. Schwann cells were infected with viruses expressing different shRNAs for either *Phb1* or *Phb2*. All shRNAs reduced expression of *Phb1* or *Phb2*, as shown by western blot (Fig. 5a,b). Schwann cells silenced for Prohibitins still extended pseudopods in response to neuronal membranes (Fig. 5c). However, when silenced Schwann cells were seeded on dorsal root ganglia neurons, fewer of them attached to neurons (Fig. 5d–f) and those that were attached frequently had an aberrant morphology on axons (Fig. 5g). This may suggest that silencing of Prohibitins in Schwann cells impairs early interactions with axons.

Because Prohibitin silencing reduced the number of Schwann cells that attached to axons, we asked whether this was due to changes in apoptosis or proliferation. Prohibitins were originally described as inhibitors of cell proliferation, hence their name^38^, and regulators of apoptosis (reviewed in ref. 39). Silencing of Prohibitins did not affect Schwann cell proliferation, but slightly increased apoptosis early after plating on axons (1 day, Supplementary Fig. 6c,d), and more significantly when Schwann cells were cultured alone (Supplementary Fig. 6a,b). After 3 days of co-culturing with axons, Schwann cell apoptosis returned to normal levels, even if the number of Schwann cells remained decreased by about 15% (Supplementary Fig. 6 e–h). This suggests that axons provide survival signals that compensate for Prohibitin silencing.

**Figure 6:**
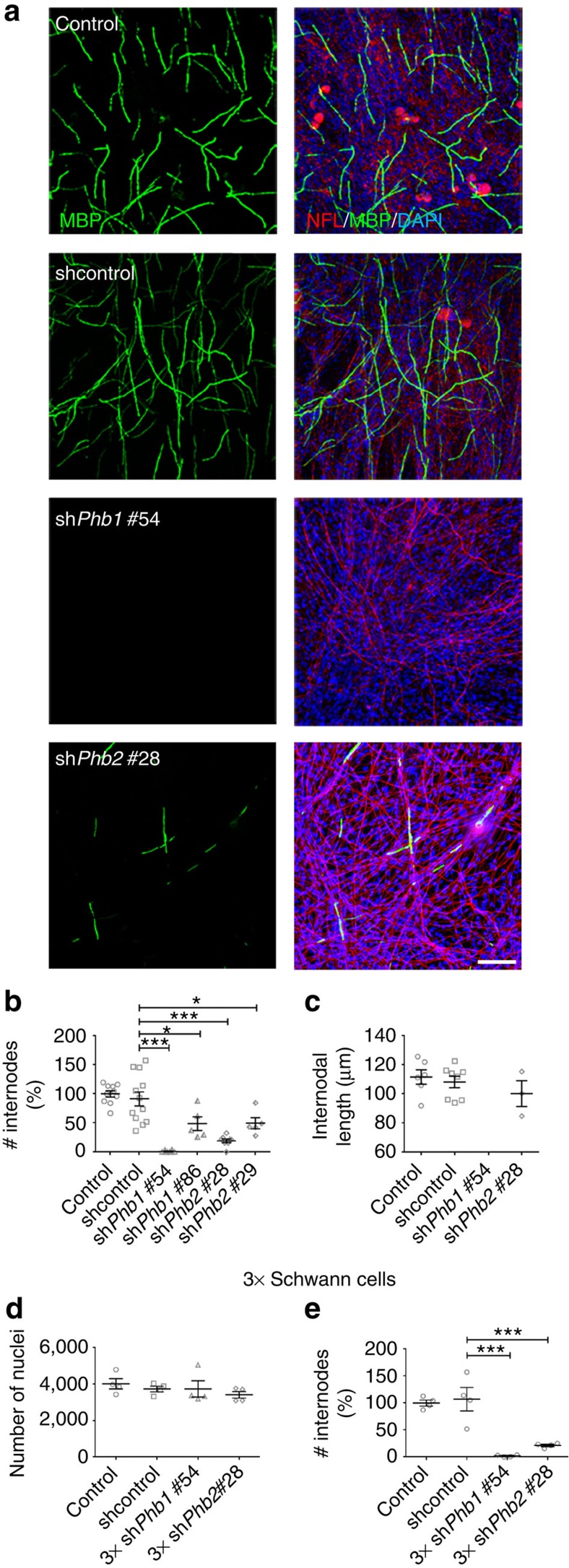
Prohibitins are required for myelination *in vitro.* (**a**) Schwann cells silenced for either *Phb1* or *Phb2* have reduced myelination. Schwann cells (200,000) were seeded on dorsal root ganglia neurons and allowed to myelinate for 7 days. In this condition 85% of the silenced cells survive (Supplementary Fig. 6h). Cultures were stained for Myelin Basic Protein (MBP, green), neurofilament (red) and DAPI (blue). The number (**b**) and length (**c**) of internodes were quantified. No myelin segments were observed when silencing for Prohibitin-1 (sh*PhB1*#54). (**d**,**e**) Three times more Schwann cells (600,000) were seeded on dorsal root ganglia neurons and allowed to myelinate for 7 days. In this condition, a similar number of control and silenced Schwann cells are present, but the defect in myelination persists. Error bars indicate s.e.m. *n*=3 coverslips from three independent experiments in **b**,**c**,**e**. Statistical analyses were performed using one-way ANOVA. *P< 0.05, ***P<0.001, Scale bar, 100 μm.

When switched to a myelinating condition by adding ascorbic acid to the cultures, myelination of axons was severely impaired after silencing of either Prohibitin-1 or -2 (Fig. 6a-c). To ask whether this was due to the reduced number of Schwann cells (15% less in this condition, Supplementary Fig. 6h) we seeded three times the amount of silenced Schwann cells on DRG neurons. As shown in Fig. 6d and e, this rescued the difference in Schwann cell number, but not myelination, indicating that the reduced number of myelin segments is not due simply to a reduction in the number of Schwann cells. Taken together, these data show that Prohibitins may be required for Schwann cell–axon interactions and that they are required for myelination *in vitro*.

### Prohibitin-2 is required for radial sorting and myelination *in vivo*

**Figure 7:**
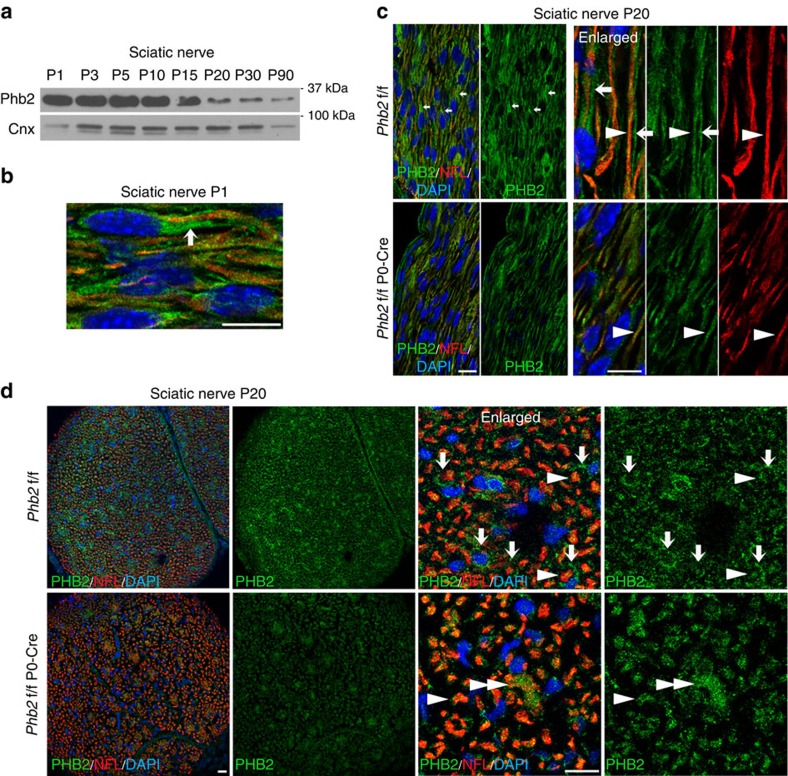
Expression of Prohibitin-2 in peripheral nerves. (**a**) Developmental expression of Prohibitin-2 in rat sciatic nerve. Protein lysates of rat sciatic nerves from P1 to P90 were blotted and probed for Prohibitin-2 and Calnexin. (**b**–**d**) Immunolocalization of Prohibitin-2 in longitudinal (**b**,**c**) and cross (**d**) sections of sciatic nerves. Sciatic nerves from mice aged P1 (**b**), and P20 (**c**,**d**) are shown. Sections were stained for Prohibitin-2 (green), Neurofilament (red) and DAPI (blue). Prohibitin-2 is detected in wild-type sciatic nerve Schwann cells (arrows) and axons (arrow heads), but it is not detectable in mutant Schwann cells. Note that Prohibitin-2 is also enriched in bundles of neurofilaments-positive axons in mutant sciatic nerves (double arrow head). Scale bars, 25 μm (**b**–**d**; left panels) and 10 μm (**c**,**d**; enlarged panel).

**Figure 8:**
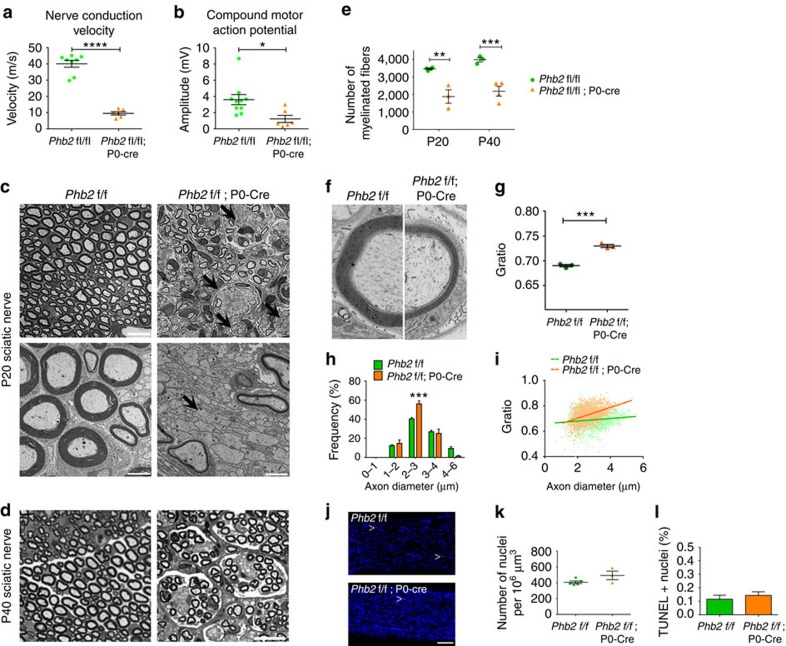
Prohibitin-2 is necessary *in vivo* for radial sorting and myelination. Schwann cell-specific deletion of Prohibitin-2 impairs radial sorting and myelination. (**a**,**b**) Electrophysiological analysis of *Phb2* f/f and *Phb2* f/f; P0-Cre mice at P20 revealed decreased nerve conduction velocity, indicative of dysmyelination (**a**, *P*<0.0001) and decreased amplitude of compound muscle action potentials, suggesting axonal pathology (**b**, *P*<0.05) in mutant sciatic nerves. (**c**) Toluidine blue stained semithin (top panel) and electron micrographs (bottom panel) cross-sections of sciatic nerves from *Phb2* f/f and *Phb2* f/f; P0-cre animals at P20. Immature bundles of unsorted axons (arrows) and few myelinated fibers are present in mutant nerves. (**d**) Toluidine blue semithin cross-sections of sciatic nerves from *Phb2* f/f and *Phb2* f/f; P0-cre animals at P40. Scale bars, 10 μm (semithins) and 2 μm (electron micrographs). (**e**) Number of myelinated axons at P20 and P40 in *Phb2* f/f and *Phb2* f/f; P0-Cre mice. (**f**) Electron micrographs show that myelinated axons of similar diameters are ensheathed by thinner myelin in mutant nerves. Scale bar, 500 nm. (**g**) G ratio was increased from 0.69 (*Phb2* f/f) to 0.73 (*Phb2* f/f; P0-Cre). (**h**) Distribution of axonal diameters in *Phb2* f/f and *Phb2* f/f; P0-Cre mice at P20 and (**i**) G ratio as a function of axon diameter. Large fibers are more hypomyelinated than small fibers. Also, there are less large fibers in *Phb2* f/f; P0-Cre mice. (**j**–**l**) TUNEL analysis on longitudinal section of sciatic nerves from *Phb2* f/f and *Phb2* f/f; P0-cre animals at P20. (J) TUNEL staining (red, white head arrows) and DAPI (blue). Scale bar, 50 μm. (**k**) Number of nuclei per 10^6^ μm^3^ of sciatic nerve. (**l**) Relative number of TUNEL positive nuclei. Error bars indicate s.e.m. *n*=8 animals in **a** and **b**; *n*= 3 animals in **e**,**g**,**h**,**k**,**l**. Statistical analyses were performed using *t*-test. *P <0.05, ***P<0.001, ****P<0.001.

Prohibitin-2 was highly expressed in sciatic nerves during active myelination (P1-P15, Fig. 7a), and its expression was shown to be modulated after nerve injury^40^. By immunohistochemistry of sciatic nerves, Prohibitin-2 appeared to localize in Schwann cells and axons, and not to be restricted to mitochondria (Fig. 7). At Postnatal day (P)1, the beginning of myelination, Prohibitin-2 staining was in Schwann cells near axons (Fig. 7b). We generated mice in which Prohibitin-2 was deleted specifically in Schwann cells. Constitutive ablation of *Phb2* is lethal *in utero* before embryonic day 9.5 (E9.5)^41^. Mice with the *Phb2* gene flanked by LoxP sites^42^ were crossed with P0-Cre mice^43^ that drive Cre expression in Schwann cells starting at E13.5^21^. In wild-type (WT) nerves Prohibitin-2 was present in Schwann cells and axons, but in mutant nerves it was only in axons at P1 and P20 (Fig. 7c,d), confirming early and Schwann cell-specific Cre-mediated recombination. Strikingly, ablation of Prohibitin-2 caused a severe motor deficit and muscular atrophy in mice at P40 (Supplementary Movie 2), due to a hypomyelinating neuropathy with a severe decrease in nerve conduction velocity (Fig. 8a). At P20 and P40, cross sections of sciatic nerves from WT littermates showed numerous myelinated axons. In contrast Prohibitin-2 deficient nerves had few myelinated axons (Fig. 8c–e), with thin myelin sheaths, as documented by an increase in their g-ratio (Fig. 8f,g). A reduction in axonal caliber (Fig. 8h,i) and in compound motor action potentials (Fig. 8b) was also observed. There was no increase in apoptosis (Fig. 8j–l). Notably, large caliber axons that were not myelinated were grouped in immature bundles (Fig. 8c), the hallmark of a defect in axonal sorting^44^, and a sign of impaired Schwann cell–axonal interactions. These results indicate that Prohibitin-2 is required both for the proper sorting of axons by Schwann cells and for their myelination *in vivo*.

## Discussion

Here we show that neuronal cell membranes induce the formation of polarized protrusions in glial cells *in vitro*, mimicking juxtacrine signalling. Using neuronal membranes in the pseudopod fractionation system, we could separate and profile these polarized glial protrusions that interacted with neuronal membranes. Nearly 10% of proteins that we have identified are implicated in glia–axonal interactions and myelination *in vivo*, validating the system. In addition, neuronal membranes induced the redistribution of a unique and novel set of proteins in Schwann cells. Thus, neuronal membranes can be used as stimuli to study juxtacrine interactions with other neuronal or glial cells. For example, it has been hypothesized that the proteins that we chose to characterize, Prohibitins, function also in neuron–neuron interactions at synapses, and may be dysfunctional in schizophrenia^45^. More broadly, the concept of isolating the portion of a cell touching another cell can be extended to any system, to study transient juxtacrine interactions, cell–cell junction formation or non-cell autonomous mechanisms in neurodegenerative disorders.

The analysis of the proteome in pseudopods begins to illuminate some of the unique events that are triggered in Schwann cells by neuronal signals. Overall, the interaction with neuronal membranes caused a change of twofold in 16% of the pseudopod proteome (Fig. 3b, Supplementary Data 2). It is possible that this change could have been even more extensive in the presence of a second polarizing cue form Laminins in the basal lamina, which is necessary for proper Schwann cell–axon interactions. The pathway and interactome analysis suggest that, in addition to anticipated roles for signalling related to cytoskeleton and G-protein coupled receptors, local protein translation, sumoylation and metabolic control are crucial at the beginning of myelination.

Cytoskeletal signalling molecules were prominent in pseudopods induced by neuronal membranes. They included regulators of Rho GTPases, Ephrins, axonal guidance molecules and Tec Kinase signalling components, supporting the idea that the cell protrusions extended by myelinating glia has the molecular characteristic of a growth cone or lamellipodia of migrating cells^6^,^46^. Subsequent myelin wrapping also requires actin polymerization and depolymerization^47^,^48^,^49^,^50^. Similarly, G-protein-related signalling was highly represented, supporting an emerging role for G-protein-coupled receptors in myelination^29^. Of note, many of these molecules were connected in protein–protein interaction networks, which also contained novel proteins, suggesting that the latter may also function in myelination or be involved in inherited human neuropathies.

Our data reveal that local control of protein expression accounts heavily for Schwann cell polarization after axonal contact. Interaction with neuronal membranes modulated the expression of molecules that regulate protein translation and degradation. Also, seven ribosomal proteins, two tRNA ligases, one elongation factor and three proteins associated with mRNA splicing were enriched in pseudopods (together accounting for 14% of the proteins enriched in pseudopods), suggesting either that local RNA regulation and translation is important for the extension of Schwann cells pseudopods or that these proteins serve extraribosomal functions.

In addition, interactome analysis revealed that post-translational modifications, in particular by SUMO, might be important in axo-glial interactions. SUMO-2 was not only redistributed in pseudopods after axonal contact, but is also known to bind or regulate >10 of the other enriched proteins (Fig. 3b), suggesting that sumoylation is an important event in myelination.

Another set of functionally related proteins that was enriched in polarized pseudopods contained enzymes involved in amino-acid metabolism including GOT1, GOT2 and malate dehydrogenase 1 (MDH1), which control levels of aspartate and glutamate, suggesting further mechanisms in neuroprotection^1^.

We identified the novel family of Prohibitins in pseudopods, and show that they are required for early axo-glial interactions. Indeed, both Prohibitin-1 and -2 are required for myelination *in vitro*, and deletion of Prohibitin-2 in Schwann cells impairs radial sorting, the process in which Schwann cells segregate large caliber axons that are destined for myelination. Prohibitin-2 may participate locally in the formation of lamellipodia-like polarized protrusions, similar to integrins and cytoskeletal regulators^5^,^6^,^21^, or in the transduction of axonal signals^12^,^51^. This latter function could occur in different Schwann cell compartments, because Prohibitins can function in various cellular location, (reviewed in refs 39 and 52) where they interact with specific protein and lipids^33^,^53^. In Schwann cells Prohibitin-2 appear to localize at the plasma membrane and mitochondria. The identification of Prohibitin interactors at the plasma membrane will reveal if Prohibitin-2 regulate signalling molecules required for myelination such as GPCR proteins, mTOR, integrins or RhoGTPases.

In T-cells, Prohibitins translocate to the plasma membrane on activation^54^ and binds to Siglec-9 on other immune cells^37^. We showed that Prohibitin-2 interacts with Schwann cells membrane proteins, and increased in pseudopods after induction with neuronal membranes, suggesting that axons may induce a redistribution of Prohibitin to pseudopods to promote Schwann cell–axon interactions. Siglecs are immunoglobulin adhesion molecules, like myelin-associated glycoprotein (or Siglec-4), thus it will be important to determine whether axons contain any Siglec molecule on their surface. Finally, Prohibitins are present in oligodendrocytes^55^ suggesting that they might be important also for central nervous system myelination.

In conclusion, we describe an innovative, modified method to study polarized cellular domains, and we used it for modeling and molecular profiling of the advancing glial sheath and to identify novel molecules involved in the interaction between glial cells and axons. The validation *in vivo* of one of these novel proteins, Prohibitin-2, demonstrates the validity of the technique for the study of cell–cell interactions.

## Methods

### Pseudopod assay

To induce pseudopods, cells were first starved for 12 h, then trypsinized with 0.5% Trypsin–EDTA, pelleted and resuspended in serum-free medium. Cells (1.5 × 10^6^) were then plated onto the upper compartment of the Boyden chamber insert. We used 4.7 cm^2^ polycarbonate transwell permeable inserts with 3-μm pores (Corning #3414) coated with 10 μg ml^−1^ Poly-L-Lysine (Sigma). Cells were maintained in DMEM, 2 mM L-Glutamine and 0.5% bovine serum albumin (BSA) for 4 h at 37 °C to attach and spread on the upper surface of the insert. Next, inserts were washed with PBS and transferred to a six-well plate containing either 200 μl of DMEM alone (no stimulus), or DMEM with either 100 ng ml^−1^ LPA, or 10 ng ml^−1^ NRG1 EGF-like domain (sNRG), neuronal or non-neuronal membranes. In other experiments the six-well plates contained MSC, or neurobasal media (Gibco) with rat dorsal root ganglia neurons plated on the bottom of the plate. For some of the experiments requiring induction with neuronal membranes, we modified the Corning six-well plates (Corning #3335) to reduce the distance between the insert and the bottom of the plate to ∼0.2 mm. This reduced the amount of primary neurons required, but did not changed the induction of pseudopods. Pseudopods were allowed to grow for 2 h at 37 °C. To initiate pseudopods retraction, the attractant in the bottom chamber was removed and replaced by serum-free DMEM. For Laminin-111 haptotactic stimulation, the inserts were first coated with Poly-L-Lysine on the top, then they were turned upside down and coated with 10 μg ml^−1^ of Laminin-111 (Sigma P5899) on the bottom.

### Preparation of neuronal membranes

Neuronal membranes were prepared from DRG of rat embryos at 15 gestational days as described in ref. 56. Briefly, 20 DRGs were dissected and plated on 9.5-cm^2^ dishes in C media: MEM (Gibco), 10% foetal bovine serum, 4 g l^−1^ D-Glucose, 2 mM L-Glutamine, 50 ng ml^−1^ Nerve Growth Factor (Harlan). After 12 h cells were switched for 2 weeks in NB medium (Gibco), 1 × B27 supplement (Gibco), 4 g l^−1^ D-Glucose, 2 mM L-Glutamine, 50 ng ml^−1^ Nerve Growth Factor (Harlan). To obtain neuron-only cultures, the cells were treated with FUDR (10 μM fluorodeoxyuridine (FdU) and 10 μM uridine) in NB medium for three cycles (2 days with FUDR, 2 days without FUDR). Neurons were collected by scraping the network with forceps and then they were homogenized in PBS with a Dounce homogenizer. The homogenized solution was centrifuged at 300 *g* for 20 min to remove debris and collagen. The supernatant was centrifuged at 30,000 *g* for 1 h. The pellets were resuspended in DMEM (67 μl for each 9.5-cm^2^ dish) and stored at −80 °C for up to 6 months. To test their activity, membranes were centrifuged onto serum starved, rat primary Schwann cells, incubated at 37 °C for an additional 20 min, lysed and analysed by western blotting. CHO and COS-7 membranes were prepared as above using two 10-cm^2^ dishes of confluent cells as starting material.

### Immunofluorescence and immunohistochemistry

For staining and imaging of filter membranes after the induction of pseudopods, Schwann cells on the top chamber and pseudopods on the bottom chamber were sandwiched between two drops of PBS and parafilm, washed twice in PBS and then stained. For phalloidin staining, Schwann cells were fixed with 4% ice-cold paraformaldehyde for 30 min, washed twice with PBS and incubated in quenching solution (75 mM NH_4_Cl; 20 mM Glycine) for 10 min at room temperature. Cells were then permeabilized with 1.6‰ Saponin in 1% BSA for 15 min at 37 °C and incubated for 1 h at 37 °C with phalloidin-TRITC 1/200 (Sigma, P1951). For immunostaining, Schwann cells were fixed in DOTMAC 0.25%, PFA 4%, washed in PBS, blocked for 1 h in 10% NGS, 3% BSA, 0.1% Triton X-100, 0.05% Tween-20 in 1X PBS and incubated overnight with the following antibodies: BD Pharmingen anti-β1 Integrin 1/100 (553837), Proteintech anti-Phb2 1/100 (12295-1-AP). Filters were then incubated for 1 h with Jackson DyLight 488 1/1,000 or 649-conjugated 1/1,000 secondary antibodies. Filters were next excised from the insert with microscissors and mounted on a slide with Vectashield (Vector Laboratories). Images were acquired with a confocal microscope Leica SP5II. Progressive Z-sections were acquired starting from the upper chamber (above the cell bodies), through the filter membrane, and in the bottom chamber (pseudopod area), and either analysed separately, or stacked in Z-projection that included only one of the compartments (that is, cell body, filter membrane or pseudopod). Three-dimensional reconstructions were performed with Fiji (fiji.sc).

Conventional immunostaining of Schwann cell–neuron co-cultures was performed with the following antibodies: Millipore anti-NFH 1/700 (AB1989) and Covance anti-MBP 1/1000 (SMI-99P) and Jackson DyLight 488 1/1,000 or 549-conjugated 1/1,000 secondary antibodies. Images were acquired with a fluorescent microscope Leica DM6000B. Myelination *in vitro* was evaluated from four different experiments, performed with two to four coverslips in each case, which is a standard sample size for these experiments. Myelin segments number and length were quantified using Image J software (http://imagej.nih.gov/ij) from two random fields of each culture at the 10 × objective. Data were analysed using Graph pad Prism 6.01. For immunocytochemistry, Schwann cells were fixed in DOTMAC 0.5%, PFA 1%. Coverslips or slide were washed in PBS, blocked for 1 h in 10% NGS, 3% BSA, 0.1% Triton X-100, 0.05% Tween-20 in 1X PBS, then incubated overnight with the following antibodies: Proteintech anti-Phb2 1/100 (12295-1-AP), Covance anti-NFH 1/700 (PCK-593P). For immunohistochemistry, sciatic nerve sections were permeabilized with acetone. Sections were rinsed in PBS, incubated 1 h with Jackson DyLight 488 1/1,000 or 549-conjugated 1/1,000 secondary antibodies, stained with DAPI, and mounted with Vectashield (Vector Laboratories). Images were acquired with a confocal microscope Leica SP5II. Maximum intensity projection was created with Fiji (fiji.sc).

### Protein extraction, western blot and antibodies

To extract proteins after the pseudopods assay, Schwann cells plated on filters with pseudopods on the lower side were washed twice in PBS, fixed with ice-cold methanol for 30 min on ice and washed again in PBS. For each filter, either the cell bodies on the upper surface or the pseudopods on the undersurface, were removed with a cotton swab. The remaining pseudopods or cell bodies were scraped into lysis buffer (100 mM Tris pH 7.4, 5 mM EDTA, 150 mM NaCl, 1% SDS, 1 mM sodium orthovanadate, protease inhibitors) and protein quantities were determined using microBCA assay (Thermo Scientific). Proteins were quantified from at least three different experiments. Data were analysed using Graph pad Prism 6.01.

Western blot was performed as described^57^. To control for equal loading of pseudopod and cell body fractions, blots were stained with Amido Black (Sigma). Blotted membranes were probed overnight with Abcam anti-Histone 2B 1/1,000 (ab1790), anti-ErbB2 1/250 (ab2428), anti-ErbB3 1/250 (ab34641); Cell Signalling anti-Akt 1/1,000 (#9272), anti-p-Akt 1/1,000 (#9271), anti-Fak 1/500 (#3283), anti-p-Fak 1/500 (#3285), anti-Erk 1/1,000 (#9102), anti-p-Erk 1/1,000 (#9101); Millipore anti-Rac1 1/200 (05-389), anti-α5 Integrin 1/250 (ab1928), anti-Prohibitin-2 1/250 (ab10198), anti-Par3 1/500 (07-330); Proteintech anti-Contactin-1 1/200 (13843-1-AP), Sigma anti-Calnexin 1/3,000 (C4731), anti-Tubulin ½,000 (T4026); Novocastra anti-β-Dystroglycan 1/40 (Ncl-b-DG); BD Bioscience anti-*N*-Cadherin 1/500 (#610920), anti-β1 Integrin 1/100 (#610468); Thermo Scientific anti-Prohibitin-1 1/250 (II-14-10), anti-Pdha1 1/1,000 (gift from Dr Patel, University at Buffalo), anti-Necl4 1/1,000 and anti-Caspr 1/1,000 (gifts from Dr Peles, Weizmann Institute). Membranes were then rinsed in PBS and incubated for 1 h with secondary antibodies. Blots were developed using ECL, ECL plus (GE Healthcare) or the Odyssey CLx infrared imaging system (Li-Cor). Western blots were performed at least two times. Uncropped blot are shown in Supplementary Fig. 7.

## Additional information

**How to cite this article:** Poitelon, Y. *et al*. Spatial mapping of juxtacrine axo-glial interactions identifies novel molecules in peripheral myelination. *Nat. Commun.* 6:8303 doi: 10.1038/ncomms9303 (2015).

## Supplementary Material

Supplementary Information Supplementary Figures 1-7, Supplementary Methods and Supplementary References

Supplementary Data 1 SILAC mass spectrometry data from Schwann cell bodies and pseudopods induced by neuronal membranes. List of proteins identified by two independent SILAC proteomic analyses in Schwann cell pseudopods or cell bodies, after induction by neuronal membranes, as compared to the proteins present in both compartments without stimulus (DMEM only). For SILAC ratio, protein intensities were normalized by the total protein intensities of the cell body or of the pseudopod proteome.

Supplementary Data 2 Proteins more abundant in pseudopods and enriched by neuronal membranes. 95 proteins were found to be more abundant in pseudopods (Ps/CB ratio > 1.25) after induction by neuronal membranes (Ps induced/non-induced > 1). The subcellular localization of each protein was derived from UniProt subcellular location.

Supplementary Data 3 Label-free mass spectrometry data from Schwann cell bodies and pseudopods, after induction with serum and growth factors. List of proteins identified by two different label-free proteomic analyses in Schwann cell body and pseudopod, after induction by fetal calf serum and growth factors.

Supplementary Data 4 SILAC mass spectrometry from neuronal membranes and comparison with Schwann cell pseudopods induced by neuronal membranes. List of proteins identified by SILAC proteomic analysis in neuronal membranes.

Supplementary Data 5 Protein ontology analysis from proteins identified in Schwann cell pseudopods. Complete protein ontology analysis of the significant canonical pathways in Schwann cell pseudopods. 162 pathways are increased after neuronal membrane stimulation as compared to DMEM.

Supplementary Data 6 Literature supporting the protein-protein interaction predictions. References supporting the protein-protein interaction network shown in Figure 3B.

Supplementary Movie 1 Z-stack from Schwann cells extending pseudopods toward axonal membranes. Reconstruction through the z-axis of Schwann cells extending pseudopods through 3 μm pores of a microporous Boyden chamber filter in response to neuronal membranes. Note the thickness of the filter around 5-6 μm. Schwann cells were exposed to neuronal membranes for 2 h, fixed then stained with TRITC-phalloidin and DAPI.

Supplementary Movie 2 Phenotypic observation of mice after *Phb2* ablation in Schwann cells. 40 days old *Phb2* f/f; P0-Cre and *Phb2* f/f mice. The mutant animal (always in the field of view) is smaller than the wild type littermate (entering the field of view on the right), and presents paralysis of one hind limb with gait impairment, tremor and muscle atrophy, all signs of a severe peripheral neuropathy.
