# Supplementary material for: Spatial mapping of juxtacrine axo-glial interactions identifies novel molecules in peripheral myelination
Source: Nat Commun. 2015 Sep 18;6:8303. doi: 10.1038/ncomms9303 (PMC4576721; doi:10.1038/ncomms9303)
Supplement: Supplementary Information — Supplementary Figures 1-7, Supplementary Methods and Supplementary References [file ncomms9303-s1.pdf]

Supplementary Figure 1

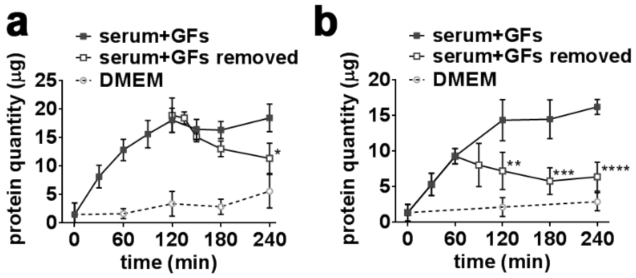

Schwann cells retract pseudopods upon removal of serum and growth factors

(A-B) The amount of proteins in pseudopods was determined for the indicated times after induction with serum and growth factor in the bottom chamber. The serum and growth factor gradient was removed after 120 min (A) or 60 (B) to allow pseudopods to retract for the indicated times. Error bars indicate s.d. n=3 independent experiments (A-B). Statistical analyses were performed using two-way ANOVA (E). * p< 0.05, ** p< 0.01, *** p< 0.001, **** p< 0.0001.

Supplementary Figure 2

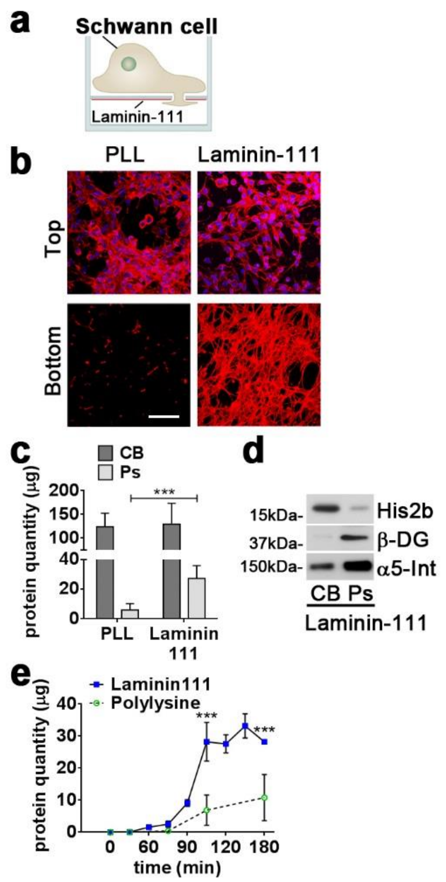

Schwann cells extend pseudopods in response to polymerized laminins (haptotactic stimulus)

(A) Schematic representation of Schwann cell pseudopod extension toward a haptotactic stimulus of laminin-111 (indicated in red) coated on the bottom side of the microporous membrane. (B) Confocal images of Schwann cell pseudopods (bottom) and cell bodies (top) in response to poly-L-lysine (PLL, negative control) or laminin-111. Cells were stained for F-actin with TRITC-phalloidin and DAPI (blue). (C) Quantification of Schwann cell pseudopod and cell body protein lysates. (D) Western blots from Schwann cell pseudopod (Ps) and cell body (CB) protein lysates, after haptotactic induction of laminin-111. Equal amounts of proteins were separated by SDS/PAGE and probed for the indicated proteins. (E) Growth kinetic of Schwann cell pseudopods. The amount of proteins was determined for the indicated times after seeding of Schwann cells onto the upper chamber. Error bars indicate s.d. n=3 independent experiments (C, E). Statistical analyses were performed using t-test (C) and two-way ANOVA (E). *** $p < 0.001$. Scale bar: 40 µm.

Supplementary Figure 3

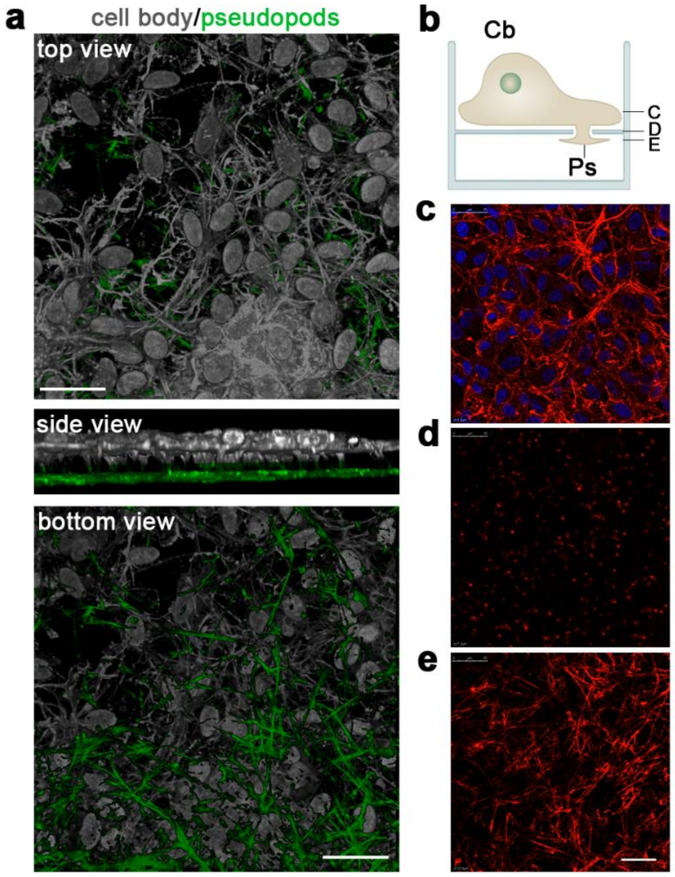

Volume rendering and confocal images of Schwann cells extending pseudopods in response to neuronal membranes

(A) 3D reconstruction and volume rendering of a confocal series of Schwann cells extending pseudopods and stained for F-actin, showing the spatial organization of the chamber and that most of the Schwann cells extend pseudopods. The cell bodies (upper side of the filter) are pseudocolored in grey and the pseudopods (bottom side of the filter) are pseudocolored in green. (B) Schematic representation of the position of the images in (C-E) on the z-axis. (C-E) Single confocal images of Schwann cell pseudopods and cell bodies in response to neuronal membranes. Cells were stained for F-actin with TRITC-phalloidin and with DAPI (blue). The related movie 1 shows movement on the z-axis from the top to the bottom chamber. Bars= 25 μm.

Supplementary Figure 4

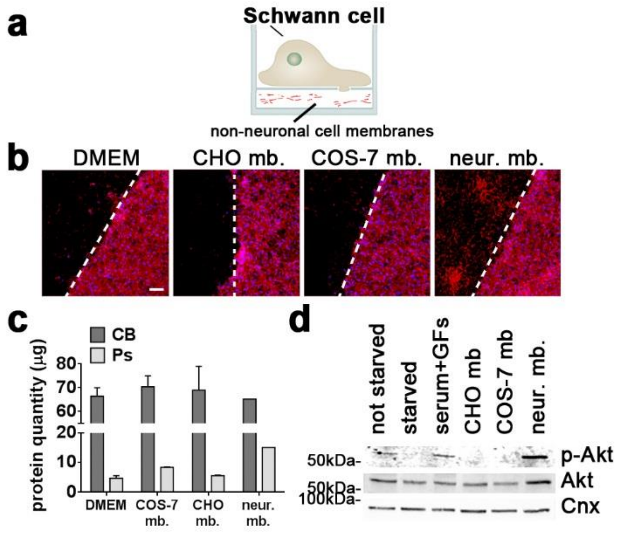

Schwann cells do not extend pseudopods in response to non–neuronal membranes

(A) Schematic representation of lack of Schwann cell pseudopods extension toward non-neuronal membranes. (B) Phalloidin-TRITC and DAPI (blue) staining of Schwann cell pseudopods and cell bodies in response to no stimulus (DMEM), CHO membranes (CHO mb), COS-7 membranes (COS-7 mb) and neuronal membranes (neur. mb). Solubilized membranes were added to the bottom chamber for 2 h. To visualize Schwann cells protrusions, half of filter on the side of the upper chamber was swabbed along the dotted line (left side). Only neuronal membranes caused the extension of polarized pseudopods. (C) Quantification by BCA of the protein content of Schwann cell pseudopods and cell bodies. (D) Lack of activation of Akt by non-neuronal membrane suspensions. Non-neuronal membranes from CHO and Cos-7 cell lines were added to starved Schwann cells, protein lysates were probed for p-Akt, Akt and Calnexin. Serum and growth factors or neuronal membranes were used as positive controls. Error bars indicate s.d. n=2 independent experiments (C). Bar= 40 µm

Supplementary Figure 5

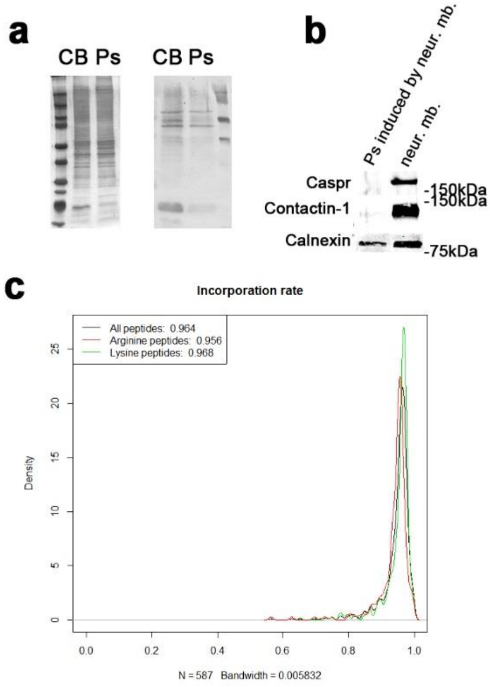

Mass spectrometry and SILAC quality controls

(A) Silver staining (left) and Amido black staining (right) shows lysates from Schwann cell bodies or pseudopods separated on SDS/PAGE. (B) Pseudopods are not contaminated by proteins from neuronal membranes. Western blot analysis for the neuronal membrane proteins Caspr and Contactin-1 shows that neither is present in Schwann cell pseudopods induced by neuronal membranes. Calnexin is used as a loading control. (C) Incorporation rate of heavy labeled Arginine and Lysine for SILAC analysis. The overall incorporation rate was 96.4%. The incorporation test was performed measuring the ratio of heavy labeled peptides to the remaining unlabeled ones. In the figure we reported the probability density function analysis obtained by kernel density estimation using the R software package.

Supplementary Figure 6

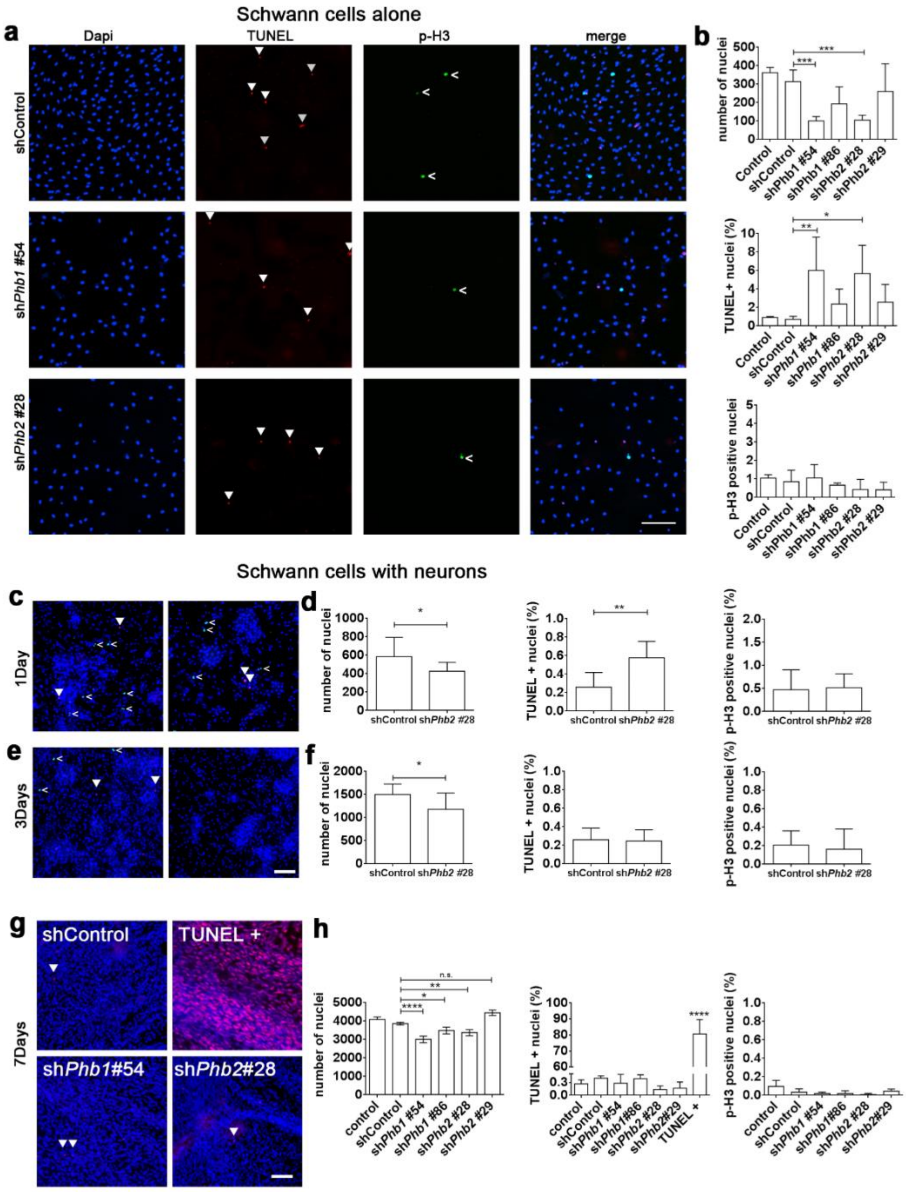

Prohibitins promote Schwann cell survival in the absence of axonal contact

TUNEL and phospho-Histone 3 (p-H3) analysis on Schwann cells silenced for either *Phb1* or *Phb2*. In panel A-B, Schwann cells were cultured alone, while in C-H, 200.000 Schwann cells were plated on neurons for different times: 1 day (C-D), 3 days (E-F) and 7 days (G-H). Co-cultures were treated with ascorbic acid. (A,C,E,G) TUNEL staining is in red, arrowheads; P-H3 staining is in green, arrows; and DAPI is in blue. (B, D, F, H): Relative number of TUNEL or p-H3 positive nuclei per field. Error bars indicate s.d. (B, D, F) or s.e.m. (H). n=3 coverslip from 3 independent experiments in B, D, F, H. Statistical analyses were performed using t-test (D, F) or

one-way ANOVA (B, H). *p<0.05, ** p<0.01, *** p<0.001, **** p<0.0001. Scale bars: 100 µm in A and 50 µm in E, G.

Supplementary Figure 7

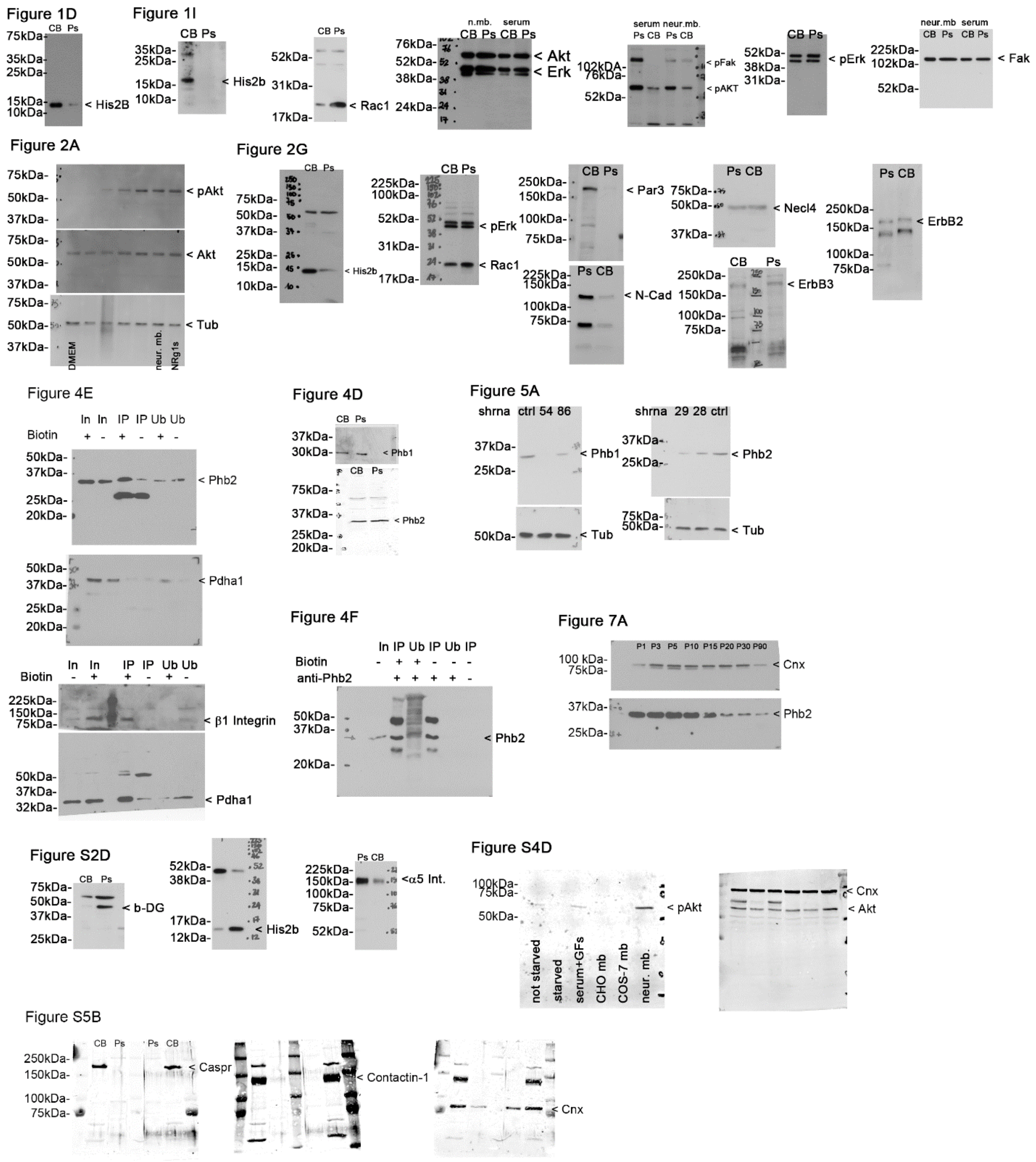

Uncropped pictures of the Western Blots shown in the manuscript

SUPPLEMENTARY METHODS

Cell cultures

Primary rat Schwann cells were prepared from sciatic nerve at P3 as described[1]. Schwann cells were maintained in Schwann cell medium (MSC): DMEM (Gibco), 1% FCS, 2 mM L-Glutamine, 2 µM Forskolin (Calbiochem), 2 ng ml^{-1} Recombinant Human NRG1-beta 1 (R&D) and passed not more than 3 times. CHO, Cos-7 and NIH3T3 cells were maintained in DMEM (Gibco), 10% FCS, penicillin and streptomycin.

MitoTracker and CellTracker

Schwann cells were incubated for 45 min with 0.2 µM of MitoTracker™ (Invitrogen, M7512) then washed with PBS. Cells were trypsinized and transferred onto the Boyden Chamber for pseudopods assay. Schwann cells were incubated for 20 min with 6.5 µM of CellTracker™ (Invitrogen, C7025) with 0.05% pluronic acid, then washed with PBS. Cells were kept for 30 min in MSC, trypsinized and plated on DRG neurons (10^5 cells/DRG) in 2 cm^2 well. After plating on DRG, cultures were incubated for 1, 3 or 24 h at 37 °C in C-media. In the 24 h condition, Ascorbic acid (50 µg ml^{-1}) was added after 8 h.

shRNA lentivirus production and infection

293T cells (NGVB, ngvbcc.org) were maintained in DMEM (Gibco), 10% FBS, 2 mM L-Glutamine, 1X MEM Non-Essential Amino Acids (Gibco, 11140-050). The media was changed 2 hours (h) prior to transfection. Cells were transfected with ViraPower™ Lentiviral Packaging Mix (Invitrogen, K4975-00) and pLKO.1 with shRNA constructs. shRNA constructs were *Phb1* shRNA (Thermo Scientific, TRCN087986 and TRCN088454) and *Phb2* shRNA (Thermo Scientific, TRCN054428 and TRCN054429). Transfection and transduction efficiencies were monitored by pLKO.1-TurboGFP™ (Sigma, SHC003). The media of 293T cells was changed 16 h after transfection, left for 30 h, and then cell supernatants were collected. The supernatants were filtered (0.22 µm), centrifuged at 30.000 g, resuspended in PBS and stored at -80 °C. Virions were titered by Q-PCR using

LV900 (ABM) kit, according to manufacturer's instructions. 0.67 millions of virions were used for each 2 cm^2 well for a transduction efficiency of 95%.

TUNEL and proliferation assays

TUNEL and proliferation assays were performed on coverslips of culture or longitudinal sections of sciatic nerves. For proliferation, cultures were fixed with 4% PFA, blocked in 20% FBS, 2% BSA, 0.1% Triton-X in PBS, and stained with anti-rabbit PH3 antibody (Millipore: #06-576), followed by Jackson DyLight 488-conjugated secondary antibody. For TUNEL positive control, coverslips were incubated 2 min in DNase buffer (0.1 mM DTT in TDT buffer: 30 mM Tris, 2 mM CoCl2, 140 mM sodium cacodylate) followed by 10 min of DNase treatment (10 U ml^{-1} of DNase I in DNase buffer). Coverslips were then incubated 15 min in TDT buffer followed by enzyme mixture (60 U ml^{-1} TdT (Sigma), 6.25 mM Biotin-16-dUTP (Roche) in TDT buffer for 1 h at 37 °C. The reaction was terminated with TB buffer (300 mM NaCl, 40 mM sodium citrate). After blocking in 2% BSA, coverslips were incubated with Streptavidin-Rhodamin secondary antibody. For longitudinal sections, slices were permeabilized 1 min in Methanol prior to TDT buffer. Nuclei were counterstained with DAPI and analyzed with fluorescence Leica DM6000B microscope. For Schwann cell-neuron co-cultures, 3 different experiments were performed after 1, 3 or 7 days of co-culture with ascorbic acid with 1-3 coverslips in each case. The proportion of pH3 and TUNEL positive nuclei was determined from 1-2 random fields of each culture at the 10x objective. For Schwann cell cultures, 2-3 different experiments were performed with 2 coverslips in each case, and the proportion of pH3 and TUNEL positive nuclei was determined from 2 random fields of each culture at the 20x objective. For longitudinal sections, 3-5 different animals per genotype were analyzed, which is a standard sample size for these experiments. The proportion or TUNEL positive nuclei were determined from 10 random fields at the 20x objective. Stained nuclei were counted manually on images using ImageJ software (http://imagej.nih.gov/ij). Data were analyzed using Graph pad Prism 6.01.

Biotinylation assay

Confluent Schwann cells were washed 3 times in PBS pH 8.0, then treated with 10 mM of EZ-Link sulfo-NHS biotin (Thermo Scientific) for 30 min at room temperature in rocking incubator. After washing 3 times with PBS containing 0.1M

glycine, cells were solubilized in modified RIPA (150 mM NaCl, 1% NP-40, 1% sodium deoxycholate, 0.1% SDS in PBS). For Streptavidin pull-down, 100 µl of Streptavidin-Sepharose (GE Healthcare) was added to each cell lysate containing the same amount of proteins, followed by stirring at 4 ºC for 16 h. For Prohibitin-2 immunoprecipitation, 1 ug of Proteintech anti-Prohibitin-2 (12295-1-AP) was added to each cell lysate containing the same amount of proteins, followed by stirring at 4 ºC for 16 h. 50ul of Dynabeads Protein G were added to the samples and incubated 4 ºC for 4 h on a stirring wheel**.** Samples were washed 3 times in modified RIPA and equal amount of proteins were separated by SDS/PAGE.

Mass spectrometry and SILAC

Mass spectrometry analysis was performed by LC-MS/MS using an LTQ-Orbitrap mass spectrometer (ThermoScientific, Bremen, Germany). Schwann cells were grown in media containing either Heavy or Light L-arginine and L-lysine (Sigma, 608033 and 608041) media for at least 5 doublings prior to pseudopod assay to allow for complete proteome labeling. To determine the incorporation rate of heavy and light aminoacids, 10µg of total lysate were loaded on SDS-PAGE. A section of the gel was digested with trypsin, and peptides were purified on StageTips and analyzed with a single LC-MS/MS run. Raw files were processed by MaxQuant analysis to determine the rate of incorporation of heavy labeled peptides. The calculation for lysine- and arginine-containing peptides was performed separately. The probability density function analysis was obtained by kernel density estimation (KDE) using the R software package (ver. 2.10.1). An overall incorporation of 96.4% was observed (Supplementary Fig. 5C). For pseudopod assay, labeled Schwann cells were placed on the Boyden chamber and pseudopod extension was induced by neuronal membranes, no stimulus (DMEM) was used as the negative control. We performed two distinct experiments and the labeling was reversed in the second experiment. Proteins were extracted, quantified using BCA and 30 µg of the samples mixed 1:1 were loaded and resolved on a gradient 7%-12% SDS-PAGE. The gel was then stained with Coomassie blue.

For Mass spectrometry and SILAC analysis, tryptic digests for each band were first cleaned using Stage Tips and then injected in a capillary chromatographic system (EasyLC, Proxeon Biosystems). Peptide separations occurred on a homemade column obtained with a 15-cm fused silica capillary (75-m inner diameter and 360 µm

outer diameter; Proxeon Biosystems) filled with Reprosil-Pur C18 3 µm resin (Dr. Maisch GmbH, Ammerbuch-Entringen, Germany) using a pressurized “packing bomb.” A gradient of eluents A (distilled water with 2% (v/v) acetonitrile, 0.1% (v/v) formic acid) and B (acetonitrile, 2% (v/v) distilled water with 0.1% (v/v) formic acid) was used to achieve separation from 8% B (at 0 min, 0.2 ml/min flow rate) to 50% B (at 80 min, 0.2 ml min^{-1} flow rate). The LC system was connected to the orbitrap equipped with a nanoelectrospray ion source (Proxeon Biosystems). Full-scan mass spectra were acquired in the LTQ-Orbitrap mass spectrometer in the mass range m/z 350 to 1500 Da and with the resolution set to 60,000. The “lock-mass” option was used for accurate mass measurements. The 10 most intense doubly and triply charged ions were automatically selected and fragmented in the ion trap. Target ions already selected for the MS/MS were dynamically excluded for 60 seconds. Protein identification and quantification was achieved using the MaxQuant software Version 1.1.1.25. Mass spectra were analyzed by Andromeda against a uniprot_cp_mus_2010_11.fasta database. The initial mass tolerance in MS mode was set to 7 ppm, and MS/MS mass tolerance was 0.5 Da. Cysteine carbamidomethylation was searched as a fixed modification, whereas N-acetyl protein and oxidized methionine were searched as variable modifications. Labeled arginine and lysine were also specified as variable modifications. SILAC peptide and protein quantification was performed automatically with MaxQuant using default settings as parameters. Protein quantification was based on extracted ion chromatograms of contained peptides. Peptide assignments were statistically evaluated using a Bayesian model on the basis of sequence length and Mascot score. Peptides and proteins were accepted with a false discovery rate of 0.01, at least two peptides identified one of which unique. All the proteomic data as raw files with relative search parameters were loaded on Peptide Atlas repository (accession number http://www.peptideatlas.org/PASS/PASS00727).

SILAC data analysis

SILAC protein ratios were calculated automatically by MaxQuant software as the median of all peptide ratios assigned to the protein. Ratio Variability was less than 30%. Systematic deviations, such as mixing errors, were corrected by the quantitation algorithm in the MaxQuant software by normalizing all peptide ratios

such that the mean of all log-transformed ratios were zero. A posterior error probability (PEP) for each MS/MS spectrum below or equal to 0.01 was required. Significant proteins were filtered using significance B with p-value<0.05 (significance B was generated by Perseus, a software included in MaxQuant package). We identified 714 proteins in Schwann cells (Supplementary Data 1). Protein intensities were normalized by the total protein intensities of cell body or pseudopod proteome. A protein was considered polarized in pseudopods if its pseudopod/cell body ratio increased by a 1.25 fold or greater, or if it was detected in pseudopods only. 176 proteins were found polarized in pseudopods and 95 pseudopod proteins were found enriched by neuronal membranes (Supplementary Data 2, Fig. 3).

Even if the pseudopods were extensively washed after induction with neuronal membranes, it is possible that some residual protein from neurons contaminated the pseudopods. To confirm that the proteins that we identified in pseudopods were not contaminants, we first compared the proteome of Schwann cell pseudopods (Supplementary Data 1), to the proteome of DRG neuronal membranes (Supplementary Data 4), and found that after induction with neurons, 43% of proteins in the Schwann cell pseudopods overlapped with the neuronal proteome, but all of these proteins (100%) were also expressed by Schwann cells that were not in contact with neurons (DMEM control). In contrast Caspr and Contactin-1, two integral membrane proteins specific of neurons, were not detectable in the pseudopods by mass spectrometry (Supplementary Data 1 and 2), or by western blot analysis (Supplementary Fig. 5B).

We performed two label free mass spectrometry analyses where pseudopod extension was promoted by serum and growth factors. Proteins from cell bodies and pseudopods fractions were extracted, quantified using BCA and 15 µg of the samples were loaded and resolved on a gradient 7%-12% SDS-PAGE. Proteins were digested as above described and Mass spectra were analyzed by Andromeda against an IPI_mouse_20100617 using MaxQuant software Version 1.1.1.2. Proteins were quantified using intensity values provided by Maxquant. A complete list of the identified proteins is found in Supplementary Data 3.

Bioinformatics

The pathway and signaling networks were analyzed using Ingenuity pathways analysis (IPA) software (Ingenuity® Systems, www.ingenuity.com, 2014). Lists of

proteins with relative enrichment in pseudopods by neuronal membrane were associated with canonical pathways in IPA. The probability of association between the dataset and the canonical pathway proteins was measured using Fisher's exact test (Supplementary Data 5). Networks of these proteins were then algorithmically generated based on their connectivity.

Statistical analyses

The data obtained are presented as mean ± s.d. or mean ± s.e.m. (for experiments performed on cells or on mice, respectively). T-test, One-way and Two-ways ANOVA with Bonferroni's multiple comparisons test were used for statistical analysis of the differences among multiples groups according to the number of samples. Values of $p<0.05$ were considered to represent a significant difference.

Animal models and morphology

All experiments involving animals followed experimental protocols approved by the San Raffaele Scientific Institute and Roswell Park Cancer Institute Animal Care and Use Committees. *Phb2* floxed mice and P0Cre transgenic mice have been described previously[3,4]. P0Cre mice were in C57BL/6 congenic background. *Phb2* fl/fl were in C57BL/6x129/SvEv mixed background. Only littermates were compared and mice of either sex were used. Genotyping of mutant mice was performed by PCR on tail genomic DNA, as described previously[3,4]. Mutant and control littermates were sacrificed at the indicated ages, and sciatic nerves were dissected. Semithin section and electron microscopic analyses of sciatic nerves were performed as previously described[5]. For g-ratio (axon diameter/fiber diameter) and axonal distribution, 4 semithin images per sciatic nerve were acquired at the 100x objective. 3 to 4 animals animal per age and per genotype were analyzed, which is the minimum number of animals required to obtain statistically significant results. Axon and fiber diameters were quantified using the Leica QWin software (Leica Microsystem). Data were analyzed using Graph pad Prism 6.01. Blinding was not possible because of the severity of the *Phb2* fl/fl; P0-Cre mice phenotype.

Electrophysiology

Phb2 fl/fl; P0-Cre and *Phb2* fl/fl mice were analyzed at 30 days of age. Mice were anesthetized with tribromoethanol, 0.02 ml g^{-1} of body weight, and placed

under a heating lamp to avoid hypothermia. Sciatic nerve motor conduction velocity and amplitude were obtained with subdermal steel monopolar needle electrodes: a pair of stimulating electrodes was inserted subcutaneously near the nerve at the ankle, then at the sciatic notch, and finally at the paraspinal region at the level of the iliac crest to obtain three distinct sites of stimulation, proximal and distal, along the nerve. Compound motor action potential were recorded with an active electrode inserted in muscles in the middle of the paw and a reference needle in the skin between the first and second digits. Blinding was not possible because of the severity of the *Phb2* fl/fl; P0-Cre mice phenotype. A minimal number of 8 sciatic nerves per genotype were analyzed, which is a standard sample size for this experiment.

REFERENCES

1. Feltri, M.L., Scherer, S.S., Wrabetz, L., Kamholz, J. & Shy, M.E. Mitogen-expanded Schwann cells retain the capacity to myelinate regenerating axons after transplantation into rat sciatic nerve. *Proc Natl Acad Sci U S A* **89**, 8827-31 (1992).
2. Pellegatta, M. *et al.* alpha6beta1 and alpha7beta1 integrins are required in Schwann cells to sort axons. *J Neurosci* **33**, 17995-8007 (2013).
3. Feltri, M.L. *et al.* P0-Cre transgenic mice for inactivation of adhesion molecules in Schwann cells. *Ann N Y Acad Sci* **883**, 116-23 (1999).
4. Park, S. *et al.* Repressor of estrogen receptor activity (REA) is essential for mammary gland morphogenesis and functional activities: studies in conditional knockout mice. *Endocrinology* **152**, 4336-49 (2011).
5. Quattrini, A. *et al.* Beta 4 integrin and other Schwann cell markers in axonal neuropathy. *Glia* **17**, 294-306 (1996).
